# Perineural Invasion Is a Significant Prognostic Factor in Oral Squamous Cell Carcinoma: A Systematic Review and Meta-Analysis

**DOI:** 10.3390/diagnostics13213339

**Published:** 2023-10-30

**Authors:** Nada Binmadi, Maha Alsharif, Soulafa Almazrooa, Suad Aljohani, Sara Akeel, Samira Osailan, Muhammad Shahzad, Wael Elias, Yasmin Mair

**Affiliations:** 1 Department of Oral Diagnostic Sciences, King Abdulaziz University Faculty of Dentistry, Jeddah 21589, Saudi Arabia; mtyalsharif@kau.edu.sa (M.A.); salmazrooa@kau.edu.sa (S.A.); sraljohani@kau.edu.sa (S.A.); yhmair@kau.edu.sa (Y.M.); 2 Department of Oral and Maxillofacial Surgery, King Abdulaziz University Faculty of Dentistry, Jeddah 21589, Saudi Arabia; 3 Institute of Basic Medical Sciences, Khyber Medical University, Hayat Abad Phase 5, Peshawar 25110, Pakistan; muhammad.shahzad@reading.ac.uk; 4 School of Biological Sciences, Health and Life Sciences Building, University of Reading, Reading RG6 6AX, UK

**Keywords:** perineural invasion, oral squamous cell carcinoma, overall survival, recurrence, meta-analysis

## Abstract

(1) Objectives: This systematic review and meta-analysis aimed to summarize current evidence regarding the prognostic role of perineural invasion (PNI) in patients with oral squamous cell carcinoma (OSCC). (2) Methods: We searched Cochrane Central, ProQuest, PubMed, Scopus, Science Direct, and Web of Science, using relevant keywords to identify eligible articles. Two independent reviewers conducted two-stage screening, data extraction, and quality assessment. The risk of bias was assessed using the Newcastle–Ottawa Scale (NOS) criteria. All analyses were performed using comprehensive meta-analysis (CMA; version 3.3.070) software. (3) Results: The study included 101 published articles encompassing 26,062 patients. The pooled analyses showed that PNI was associated with significantly worse overall survival (OS; HR = 1.45, 95% CI: 1.32–1.58; *p* < 0.001), worse disease-specific survival (DSS; HR = 1.87, 95% CI: 1.65–2.12; *p* < 0.001), and worse disease-free survival (DFS; HR = 1.87, 95% CI: 1.65–2.12; *p* < 0.001). Similarly, both local recurrence-free survival (LRFS) and regional recurrence-free survival (RRFS) were worse in patients with PNI (HR = 2.31, 95% CI: 1.72–3.10, *p* < 0.001; and HR = 2.04, 95% CI: 1.51–2.74, *p* < 0.001), respectively. The random-effect estimate of three studies demonstrated that the presence of PNI was associated with worse failure-free survival (FFS; HR = 2.59, 95% CI: 1.12–5.98, *p* < 0.001). (4) Conclusions: The current evidence suggests that PNI can be used as an independent predictor of the prognosis for patients with OSCC. The presence of PNI was associated with worse OS, DFS, DSS, FFS, and with recurrence. Asian patients and patients with extra-tumoral or peripheral PNI invasion were associated with worse prognosis.

## 1. Introduction

Oral squamous cell carcinoma (OSCC) is also known as oral cancer. It is the 16th most common type of cancer across the globe and constitutes around 95% of head and neck cancers [1,2]. While the prognosis of OSCC is generally poor [3,4], that of some subtypes, such as oral tongue SCC (OTSCC), are even worse [5,6]. One of the many possible reasons for poor prognosis among cancer patients is metastasis, which is the invasion and spread of cancerous cells to other sites in the body than from where it originated. One such route of cancer spread/metastasis is via the nervous system, a process known as perineural tumor growth. PNI is characterized by the presence of tumor cells around one-third of the nerve or the presence of tumor cells inside the epineurium, perineural space, or nerve sheath, and is usually assessed vi the histological examination of tissues [7]. PNI is a common occurrence in many types of cancers, including cervical (9–31%), colorectal (16–39%), head and neck (5.2–90%), prostate (12–84%), biliary tract tumors (56–88%), gastric (7–76%), and pancreatic cancer (70–100%) [8]. With the exception of prostate cancer, where the PNI is linked to locoregional recurrence, PNI is independently related to a worse prognosis and shorter survival in all of these other malignancies [8]. Leibig et al. and others have characterized PNI in head and neck cancer as neoplastic cells infiltrating the perineurium layer, tracking through nerves, and/or enclosing at least one-third of the nerve’s circumference [7,9]. Head and neck squamous cell carcinoma (HNSCC) patients with PNI are more likely to have poor outcomes, thereby necessitating adjuvant treatment modalities [10,11]. Similarly, in oral cancer, PNI is a significant predictor of a poor prognosis, and its presence is considered a clinical indication for radiotherapy and systemic treatment [6,12,13,14,15]. Elective neck dissection, especially for stage 1 and 2 diseases, may be required because of the association between PNI and OTSCC depth of invasion [16,17]. Incorporating PNI into OTSCC staging systems has been recommended by several studies [18]; nonetheless, there are still significant discrepancies in their findings. It is common practice for pathologists to document PNI, and its presence may have implications for how patients are treated. Therefore, this systematic review and meta-analysis is aimed at summarizing the available evidence on the prognostic role of PNI in patients with OSCC.

## 2. Materials and Methods

For a systematic review of interventions, we used the Preferred Reporting Items for Systematic Reviews and Meta-Analyses (PRISMA) checklist and the Cochrane handbook. [19,20]. We filed our systematic review and meta-analysis with the PROSPERO international prospective register of systematic reviews (registration number: CRD42022371657).

### 2.1. Eligibility Criteria

This study included studies matching the following eligibility criteria:

1. Population: Studies that included patients with OSCC irrespective of the lesion site, type, size, thickness, depth, stage, or differentiation.
2. Exposure: Studies that reported data regarding the prevalence of PNI and its type or location.
3. Comparison: Studies that compared between patients with and without PNI.
4. Outcomes: Studies that reported data regarding the association between the presence of PNI and overall survival (OS), disease-specific survival (DSS), disease-free survival (DFS), and recurrence rate.
5. Study design: Observational studies (case-control, cohort, and cross-sectional).
6. The selected articles were restricted to those published in the English language.

The exclusion criteria were as follows:

1. Case reports and conference abstracts.
2. Studies that reported/published in a language other than English.
3. In vitro studies or studies involving animal models.
4. Duplicate articles

### 2.2. Information Sources and Search Strategy

Initially, we searched Cochrane Central, ProQuest, PubMed, Scopus, Science Direct, and Web of Science databases using the following keywords “(squamous cell carcinoma OR squamous carcinoma) AND (perineural invasion OR perineural extension OR perineural infiltration) AND oral” in March 2019. The literature was further updated in 2022, to find and include more recent research studies on this topic. Databases were searched from their inception to the search date. Furthermore, all included citations’ reference lists were searched. The retrieved citations were imported and stored in a single library in EndNote X9 software, and duplicate publications were eliminated.

### 2.3. Selection Process and Data Extraction

A data collection sheet that included the research ID, publication year, title, abstract, keywords, DOI, and URL was built using Microsoft Excel. Two independent reviewers (NB and SA) conducted the selection process in two steps. In the initial step, the reviewers screened the title and abstract of all studies identified in the literature search to determine which studies would advance to the subsequent step (full-text screening), where reviewers would carefully assess whether each study fulfilled the requirements of inclusion. Any conflict between the reviewers were resolved by the third reviewer (YM).

Two reviewers (NB and SA) collected the following data from the eligible studies independently into a pre-prepared Excel spreadsheet covering different parameters, including enrolled patient demographics (age and sex), study characteristics (study groups, study date, follow-up time, total number of samples, study country, and main conclusions), lesion characteristics (type, size, location, size, thickness, depth, and surgical margins), and outcomes (PNI, recurrence, OS, DSS, and DFS). Any discrepancies were discussed and resolved by the third reviewer (YM).

### 2.4. Risk of Bias and Quality Assessment

Two writers (NB and MA) separately completed quality assessments. Discrepancies in the assessment process were handled by discussion until agreement was reached. Newcastle–Ottawa Scale (NOS) criteria were used to assess the risk of bias in the included research [21]. The Newcastle–Ottawa Scale consists of 8 items divided into 3 domains, with a maximum score of 9. A study with a score of 7–9 is deemed as good quality, 4–6 as fair quality, and 0–3 as poor quality.

### 2.5. Statistical Analyses

The DerSimonian–Laird random-effects model [22] was used for meta-analysis. Comprehensive meta-analysis (CMA; Englewood, NJ, USA: version 3.3.070) was used for statistical analyses. Fixed-effect or random-effects meta-analyses utilizing the inverse variance weighting method yielded pooled estimates of the hazard ratios (HRs), with a confidence interval (CI) of 95% based on published confidence intervals for these HRs. Using the I^2^ statistic, we calculated the percentage of the degree of heterogeneity and inconsistency among studies. The categorizing values of 25%, 50%, and 75% indicated low, moderate, and high levels of heterogeneity, respectively. If the heterogeneity was significant and I^2^ was greater than 50%, the random-effects model was used; otherwise, the fixed-effects model was used. To resolve heterogeneity, sequential sensitivity analysis was used, which involves deleting one study from each scenario. Subgroup analysis was also carried out to reduce the risk of inconsistency. Based on the parameters of Egger’s test, publication bias was assessed, and a funnel plot was created for forest plots with 10 or more studies [23]. A *p*-value of less than 0.05 was considered significant.

## 3. Results

### 3.1. Search Results

**Figure 1:**
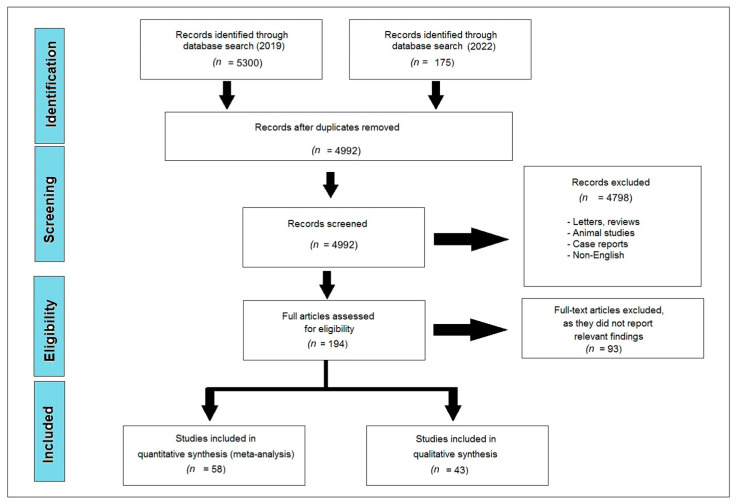
PRISMA flow diagram.

We searched six authentic databases (Cochrane Central, ProQuest, PubMed, Scopus, Science Direct, and Web of Science) and found 5475 references matching our inclusion criteria. Using Endnote software version 20.1, we eliminated duplicate references and obtained a total of 4992 research articles that were further screened. Title and abstract screening resulted in the exclusion of a further 4978 citations from our study because these studies were either published in a language other than English, or based on animal models, or were case reports, reviews, letters, or irrelevant articles. Subsequently, full-text screening was applied to the remaining studies (194 articles). Finally, we included 101 published articles that included a total of 26,062 patients, which discussed the incidence of perineural invasion among oral squamous cell carcinoma patients and its association with other co-morbidities and mortality rates [4,10,11,16,24,25,26,27,28,29,30,31,32,33,34,35,36,37,38,39,40,41,42,43,44,45,46,47,48,49,50,51,52,53,54,55,56,57,58,59,60,61,62,63,64,65,66,67,68,69,70,71,72,73,74,75,76,77,78,79,80,81,82,83,84,85,86,87,88,89,90,91,92,93,94,95,96,97,98,99,100,101,102,103,104,105,106,107,108,109,110,111,112,113,114,115,116]. Out of these studies, 43 articles were included in our qualitative analysis (systematic review) [4,11,16,26,29,30,31,32,33,37,43,44,45,47,48,52,55,56,57,59,61,62,68,72,73,75,78,80,85,86,88,89,91,92,99,101,102,103,108,109,113,115,116], and 58 articles were included in our quantitative analysis (meta-analysis) [10,18,24,25,27,28,34,35,36,38,39,40,41,42,46,49,50,51,53,54,58,60,64,65,66,67,69,70,71,74,76,77,79,81,82,83,84,87,90,93,94,95,96,97,98,100,104,105,106,107,110,111,112,114,117,118,119,120]. The study flow diagram for the study selection process is shown in Figure 1.

### 3.2. Characteristics of Included Studies

The year of publication ranged from 1995 to 2021. The majority of the published studies (*n* = 25) were reported from Taiwan, followed by India (*n* = 17), the USA (*n* = 15), China (*n* = 8), Australia (*n* = 5), Italy (*n* = 5), Brazil (*n* = 4), and three each from the UK, Germany, Israel, and the rest of the world. In terms of the study design of these included studies, 95 studies were cohort studies, 3 were case-control studies, 2 were cross-sectional studies, and 2 were case-series. The average percentage of men among the included studies was 72.31%. The range of follow-up was 1–10 years. The characteristics of included studies and patients are summarized in Supplementary Table S1.

### 3.3. Quality Assessment of Included Studies

**Figure 2:**
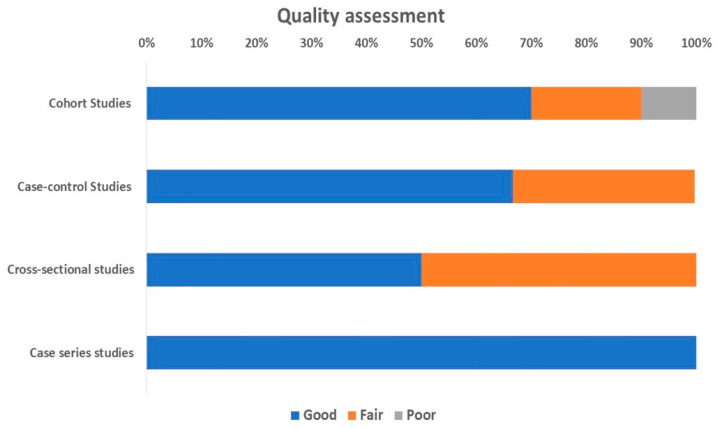
Quality assessment of included studies.

Based on the used tools, we found that 70% of the cohort studies, 67% of the case-control studies, 50% of the cross-sectional studies, and all of the case-series were deemed as “Good”. Only 10% of the cohort studies were deemed as “Poor”, as shown in Figure 2.

### 3.4. Meta-Analysis

#### 3.4.1. Overall Survival (OS)

**Figure 3:**
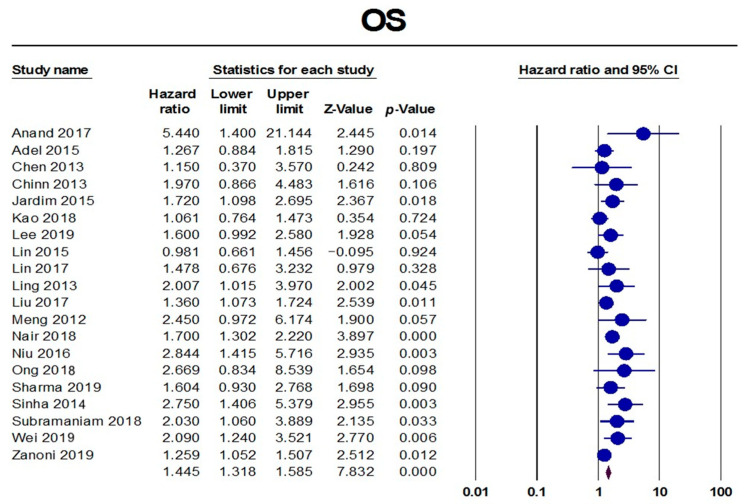
Pooled analysis of OS [10,25,28,34,35,51,53,60,65,66,67,71,74,76,77,87,90,92,104,114].

**Figure 4:**
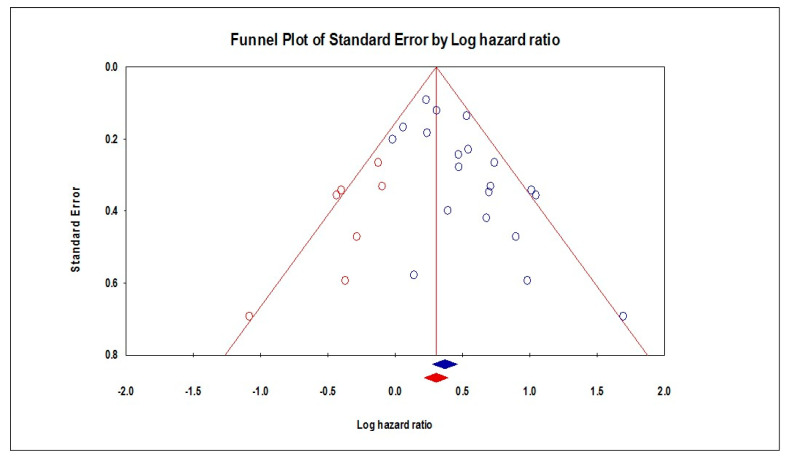
Funnel plot of OS.

The pooled analysis of HRs extracted from 20 studies showed that PNI was associated with significantly increased HRs in terms of OS (HR = 1.45, 95% CI: 1.32–1.58; *p* < 0.001), as shown in Figure 3. These pooled data were mildly heterogenous (I^2^: 37%; *p* = 0.05). We found a potential risk of publication bias (Eggers’ test *p*-value = 0.002), which could be resolved by trimming seven studies, resulting in HR = 1.35 (95% CI: 1.24–1.48), as shown in Figure 4. Subgroup analysis showed that the worst OS was found in China (HR = 2.42, 95% CI: 1.54–3.80), followed by India (HR = 1.77, 95% CI: 1.42–2.22), the USA (HR = 1.74, 95% CI: 1.02–2.96), Brazil (HR = 1.66, 95% CI: 1.12–2.45), and Taiwan (HR = 1.32, 95% CI: 1.11–1.57). Moreover, the presence of PNI was associated with worse OS in the hard palate and mandible (HR = 2.69, 95% CI: 1.54–4.70), followed by the tongue (HR: 2.06, 95% CI: 1.38–3.06), in the tongue and floor of the mouth (HR = 1.77, 95% CI: 1.20–2.63), the tongue and buccal mucosa (HR = 1.41, 95% CI: 1.03–1.93), and in the oral cavity (HR = 1.40, 95% CI: 1.19–1.63), Table 1.

#### 3.4.2. Disease-Free Survival (DFS)

**Figure 5:**
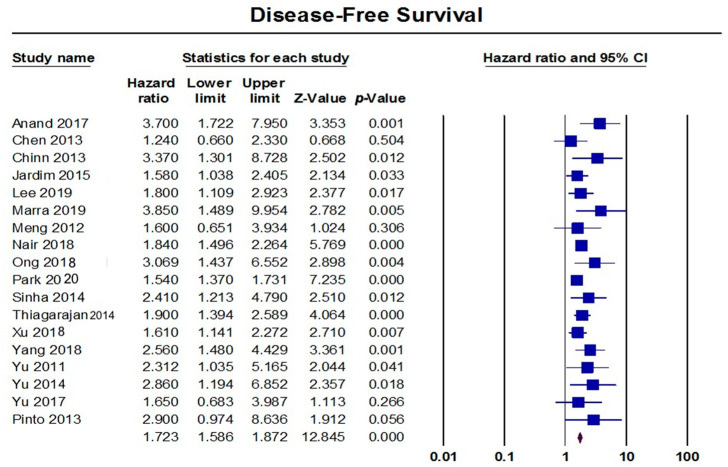
Pooled analysis of DFS [28,34,35,51,60,69,71,74,77,79,82,90,100,106,107,110,111,112].

**Figure 6:**
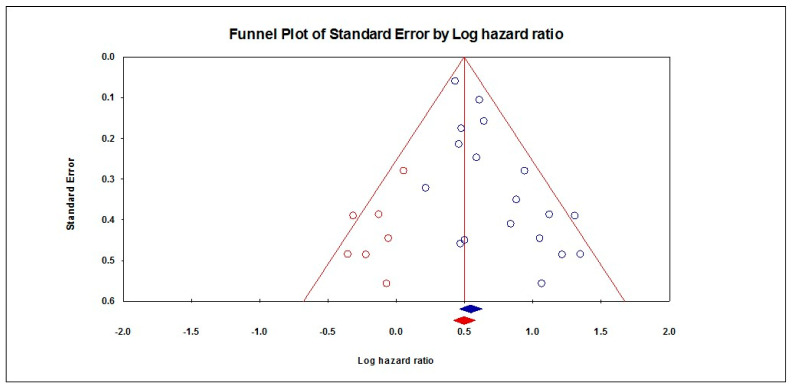
Funnel plot of DFS.

The random-effects model that included 18 studies showed a significant association between the presence of PNI and a worse DFS (HR = 1.72, 95% CI: 1.59–1.87; *p* < 0.001), as shown in Figure 5. These pooled data were homogenous (I^2^: 23%; *p* = 0.183), with a significant risk of publication bias (*p* < 0.001), as shown in Figure 6. By excluding seven studies from the analysis, the effect size adjusted to HR = 1.64 (95% CI: 1.52–1.78). Subgroup analyses demonstrated that studies from the USA reported a worse DFS (HR = 2.70, 95% CI: 1.55–4.72), followed by studies from China (HR = 1.96, 95% CI: 1.49–2.58), India (HR = 1.92, 95% CI: 1.62–2.27), Taiwan (HR = 1.77, 95% CI: 1.33–2.36), and Brazil (HR = 1.71, 95% CI: 1.15–2.53). Moreover, the presence of PNI was associated with a worse DFS when PNI occurred in the hard palate and mandible (HR = 2.60, 95% CI: 1.45–4.66), followed by the tongue (HR = 2.24, 95% CI: 1.78–2.83), in the oral cavity (HR = 1.79, 95% CI: 1.41–2.29), in the tongue and buccal mucosa (HR = 1.77, 95% CI: 1.45–2.15), and in the tongue and floor of the mouth (HR = 1.57, 95% CI: 1.40–1.75). Patients with an early tumor stage had a worse DFS (HR = 2.01, 95% CI: 1.57–2.63) compared to those with an advanced stage (HR = 1.83, 95% CI: 1.48–2.27). Regarding the location of the PNI, patients with extra-tumoral invasion had a worse DFS (HR = 2.59, 95% CI: 2.39–2.81) compared to those with peripheral invasion (HR = 2.33, 95% CI: 1.98–2.74), as shown in Table 2.

#### 3.4.3. Disease-Specific Survival (DSS)

**Figure 7:**
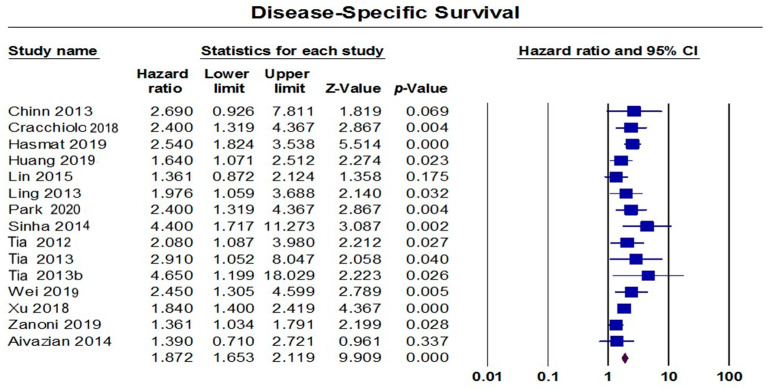
Pooled analysis of DSS [10,27,35,36,42,49,66,79,90,95,96,97,104,106,114].

**Figure 8:**
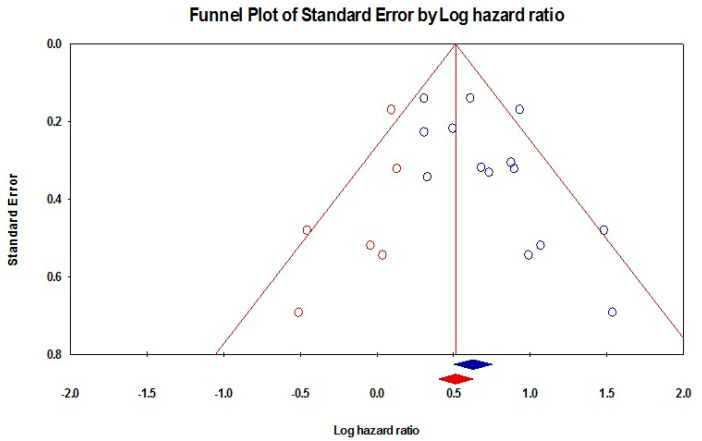
Funnel plot of DSS.

The random-effect model that included 15 studies showed a significant association between the presence of a PNI and a worse DSS (HR = 1.87, 95% CI: 1.65–2.12; *p* < 0.001), Figure 7. These pooled data were homogenous (I^2^: 29%; *p* = 0.138), with a significant risk of publication bias (*p* = 0.032), Figure 8. By trimming six studies from the analysis, the effect size was adjusted to HR = 1.67 (95% CI: 1.49–1.87). Subgroup analyses demonstrated that Australian studies reported a worse DSS (HR = 2.29, 95% CI: 1.75–2.98), followed by studies from the USA (HR = 2.20, 95% CI: 1.29–3.78), China (HR = 1.86, 95% CI: 1.45–2.40), and Taiwan (HR = 1.82, 95% CI: 1.43–2.32). Moreover, the presence of a PNI was associated with a worse DSS when PNI occurred in the tongue (HR = 2.87, 95% CI: 1.83–4.51), in the oral cavity (HR = 1.56, 95% CI: 1.40–1.94), in the tongue and buccal mucosa (HR = 1.76, 95% CI: 1.23–2.51), or in the tongue and floor of the mouth (HR = 2.31, 95% CI: 1.78–2.98). Similarly, patients with an early tumor stage were associated with a worse DSS (HR = 2.17, 95% CI: 1.62–2.93) compared to those with an advanced stage (HR = 1.83, 95% CI: 1.30–2.58). Regarding the location of the PNI, patients with extra-tumoral invasion were associated with a worse DSS (HR = 2.28, 95% CI: 1.87–2.78) compared to those with an intra-tumoral (HR = 2.06, 95% CI: 1.57–2.71) or a peripheral invasion (HR = 1.90, 95% CI: 1.10–3.25). Regarding the size of the PNI, a larger size was associated with a worse DSS compared to a smaller size (HR = 1.74, 95% CI: 1.19–2.52 vs. HR = 1.45, 95% CI: 0.81–2.60), Table 3.

#### 3.4.4. Local Recurrence-Free Survival

The pooled analysis that included 12 studies demonstrated that the presence of PNI was associated with a worse LRFS (HR = 2.31, 95% CI: 1.72–3.10, *p* < 0.001). These pooled data were heterogenous (I^2^: 50%; *p* = 0.017). By excluding two studies (Lin et al., 2015 and Hasmat et al., 2019) [42,66], this heterogeneity was resolved (I^2^: 16%; *p* = 0.287), and the effect size was significant (HR = 2.62, 95% CI: 2.03–3.38, *p* < 0.001).

#### 3.4.5. Regional Recurrence-Free Survival

The fixed-effect estimate that included four studies demonstrated that the presence of PNI was associated with a worse RRFS (HR = 2.04, 95% CI: 1.51–2.74, *p* < 0.001). These pooled data were homogenous (I^2^: 14%; *p* = 0.32).

#### 3.4.6. Failure Free Survival

The random-effect estimate that included three studies showed that the presence of a PNI was associated with a worse FFS (HR = 2.59, 95% CI: 1.12–5.98, *p* < 0.001). The pooled data were heterogenous (I^2^: 63%; *p* = 0.062). By excluding Aivazian et al., 2014 [27], this heterogeneity was resolved (I^2^: 0%; *p* = 0.484), and the effect size was HR = 3.91 (95% CI: 1.99–7.65, *p* < 0.001).

## 4. Discussion

Recently, many research studies have evaluated the role of PNI in OSCC clinical outcomes. However, these findings are still contradictory. The goal of this meta-analysis was to evaluate whether individuals with OSCC and PNI had worse prognoses compared to non-PNI cases. The overall results of our study revealed that PNI was likely to worsen OS, DFS, DSS, LRFS, RRFS, and FFS. In terms of OS, we found that a worse OS occurred in Asian countries compared to in Europe and America. These findings are not unusual since Asian countries have the highest prevalence of OSCC. According to the Global Cancer Observatory (GCO) 2020 report [121], of the total 377,713 cases of OSCC worldwide, the highest number of cases was reported in Asian countries (248,360), followed by Europe (65,279), and North America (27,469). Similar trends were also reported in a number of studies wherein most of the cases included were reported from Asian countries, and, as such, more data from Europe and America are needed to further clarify the role of a PNI in prognosis. In terms of PNI extension, the size of the nerve involved provided additional prognostic information. For example, in OSCC cases with multifocal PNI, the worst DSS was observed when the size of the nerve involved exceeded 1 mm, and a better prognosis was observed if the size was less than 1 mm. Survival was also dependent on the location of the tumor. Worse OS and DFS were associated with the presence of PNI in the hard palate and mandible, followed by in the tongue and oral cavity. Furthermore, the DSS was significantly worse among patients who had PNI and OTSCC than among those who had PNI in any other site in the oral cavity.

Our study results are in concordance with the findings of a recent meta-analysis of patients with OTSCC, wherein the presence of PNI was associated with a worse cancer-specific survival (CSS) (HR = 1.93, *p* < 0.001), a worse DFS (HR = 2.13, *p* < 0.001), a worse DFS (HR = 2.13, *p* < 0.001), and a higher risk of LRFS (HR = 1.73, *p* = 0.025). Additionally, only early-stage OTSCC was affected by PNI in terms of locoregional recurrence. However, CSS, DFS, and OS were affected in all stages of OTSCC [122]. Another meta-analysis that investigated the prognostic role of PNI in HNSCC demonstrated that PNI was significantly associated with OS (HR = 2.80, *p* < 0.001), DFS (HR = 2.42, *p* < 0.001), and DSS (HR = 2.60, *p* < 0.001) [123]. Based on our findings, the prognostic value of PNI in OSCC has been established. Patients with OSCC may benefit from more aggressive treatment if their PNI levels are elevated. Research evidence also suggests that the patients with skin, oral, and colorectal cancer should undergo PNI testing, which will help clinicians to plan better treatment and management strategies [34,36]. For example, in patients with OSCC and PNI, Yang et al. [80] found that elective neck dissection targeting macroscopic disease did not enhance the prognosis, thereby requiring adjuvant radiation and systemic therapy. However, it is too early to recommend and endorse this strategy, and further research is required.

The heterogeneities in this meta-analysis were expected and fell within the accepted limits. Since these studies were reported from different countries across the globe, there were differences in the patients’ ethnic backgrounds, and among the study periods, tumor characteristics, and treatment modalities, thereby greatly affecting the prognoses. Additionally, the consistency between the included studies might be affected by interobserver differences, the number of times a tissue section is examined, the histological sectioning method, and the size of the tissue obtained. In order to counter the issue of inconsistency among these studies, we further conducted a subgroup analysis. However, to acquire consistency and reproducibility among interobserver studies, and to minimize subjectivity, accurate identification of PNI is very important, requiring additional standardized reporting to include the diameters of the involved nerves.

Although, our study is the first of its kind in terms of meta-analyses reporting the impact of PNI on the prognosis of OSCC, there are some limitations. The number of prospective studies we included was small and there is the possibility that biases carried over from retrospective studies could have affected our findings. Another limitation is publication bias. However, after the application of trim-and-fill analysis, there was no significant change in the effect size.

## 5. Conclusions

In conclusion, the current evidence suggests that PNI can be used as an independent predictor for the prognosis of a patient with OSCC. PNI presence was associated with worse OS, DFS, DSS, and FFS, and with recurrence. Asian patients and patients with extra-tumoral or peripheral PNI invasion were associated with a worse prognosis.

## Figures and Tables

**Table 1:** Subgroup analyses of OS.

Domain	Subgroup	No. Studies	HR (95% CI)	*p*-Value	Heterogeneity
Country	USA	3	1.74 (1.02–2.96)	0.041	I^2^: 65%; *p* = 0.058
	India	4	1.77 (1.42–2.22)	<0.001	I^2^: 0.4%; *p* = 0.39
	China	3	2.42 (1.54–3.80)	<0.001	I^2^: 0%; *p* = 0.770
	Taiwan	8	1.32 (1.11–1.57)	0.002	I^2^: 25%; *p* = 0.23
	Brazil	2	1.66 (1.12–2.45)	0.011	I^2^: 0%; *p* = 0.742
Sample size	≥150	12	1.48 (1.27–1.73)	<0.001	I^2^: 44%; *p* = 0.052
	<150	8	1.74 (1.33–2.26)	<0.001	I^2^: 19%; *p* = 0.280
Site of tumor	Tongue	3	2.06 (1.38–3.06)	<0.001	I^2^: 0%; *p* = 0.425
	Oral cavity	8	1.40 (1.19–1.63)	<0.001	I^2^: 23%; *p* = 0.243
	Tongue/floor of the mouth	2	1.77 (1.20–2.63)	0.004	I^2^: 0%; *p* = 0.776
	Tongue/buccal mucosa	5	1.41 (1.03–1.93)	0.032	I^2^: 55%; *p* = 0.06
	Other **	2	2.69 (1.54–4.70)	<0.001	I^2^: 0%; *p* = 0.801
Stage of tumor	Early	4	1.56 (1.12–2.17)	0.009	I^2^: 15%; *p* = 0.315
	Advanced	6	1.52 (1.27–1.81)	<0.001	I^2^: 0%; *p* = 0.422
	Both	10	1.56 (1.26–1.92)	<0.001	I^2^: 57%; *p* = 0.012

** Hard palate and mandible.

**Table 2:** Subgroup analysis of DFS.

Domain	Subgroup	No. Studies	HR (95% CI)	*p*-Value	Heterogeneity
Country	USA	2	2.70 (1.55–4.72)	<0.001	I^2^: 0%; *p* = 0.576
	India	3	1.92 (1.62–2.27)	<0.001	I^2^: 33%; *p* = 0.224
	China	3	1.96 (1.49–2.58)	<0.001	I^2^: 43%; *p* = 0.173
	Taiwan	6	1.77 (1.33–2.36)	<0.001	I^2^: 0%; *p* = 0.718
	Brazil	2	1.71 (1.15–2.53)	0.007	I^2^: 3%; *p* = 0.309
	Other *	2	1.56 (1.40–1.75)	<0.001	I^2^: 71%; *p* = 0.06
Sample size	≥150	6	1.88 (1.64–2.16)	<0.001	I^2^: 0%; *p* = 0.597
	<150	12	1.64 (1.48–1.87)	<0.001	I^2^: 31%; *p* = 0.139
Site of tumor	Tongue	5	2.24 (1.78–2.83)	<0.001	I^2^: 0%; *p* = 0.514
	Oral cavity	5	1.79 (1.41–2.29)	<0.001	I^2^: 0%; *p* = 0.756
	Tongue/floor of the mouth	4	1.57 (1.40–1.75)	<0.001	I^2^: 21%; *p* = 0.284
	Tongue/buccal mucosa	2	1.77 (1.45–2.15)	<0.001	I^2^: 26%; *p* = 0.244
	Other **	2	2.60 (1.45–4.66)	0.001	I^2^: 48%; *p* = 0.164
Stage of tumor	Early	5	2.01 (1.57–2.63)	<0.001	I^2^: 56%; *p* = 0.057
	Advanced	5	1.83 (1.48–2.27)	<0.001	I^2^: 30%; *p* = 0.222
	Both	11	1.68 (1.54–1.84)	<0.001	I^2^: 5%; *p* = 0.396
Location of PNI	Extra-tumoral	2	2.59 (2.39–2.81)	<0.001	I^2^: 55%; *p* = 0.135
	Intra-tumoral	1	1.22 (0.47–3.16)	0.682	-
	Peripheral	1	2.33 (1.98–2.74)	<0.001	-
	Unknown	17	1.72 (1.58–1.87)	<0.001	I^2^: 27%; *p* = 0.142

* Australia and Italy. ** Hard palate and bucco/alveolar.

**Table 3:** Subgroups of DSS.

Domain	Subgroup	No. Studies	HR (95% CI)	*p*-Value	Heterogeneity
Country	USA	4	2.20 (1.29–3.78)	<0.001	I^2^: 64%; *p* = 0.039
	China	2	1.86 (1.45–2.40)	<0.001	I^2^: 0%; *p* = 0.837
	Taiwan	6	1.82 (1.43–2.32)	<0.001	I^2^: 9%; *p* = 0.353
	Australia	3	2.29 (1.75–2.98)	<0.001	I^2^: 20%; *p* = 0.283
Sample size	≥150	12	1.81 (1.59–2.06)	<0.001	I^2^: 27%; *p* = 0.172
	<150	3	2.82 (1.79–4.46)	<0.001	I^2^: 0%; *p* = 0.564
Site of tumor	Tongue	3	2.87 (1.83–4.51)	<0.001	I^2^: 0%; *p* = 0.567
	Oral cavity	6	1.65 (1.40–1.94)	<0.001	I^2^: 31%; *p* = 0.199
	Tongue/floor of the mouth	4	2.31 (1.78–2.98)	<0.001	I^2^: 0%; *p* = 0.456
	Tongue/buccal mucosa	2	1.76 (1.23–2.51)	0.002	I^2^: 0%; *p* = 0.548
Stage of tumor	Early	6	2.17 (1.62–2.93)	<0.001	I^2^: 0%; *p* = 0.508
	Advanced	2	1.83 (1.30–2.58)	0.001	I^2^: 0%; *p* = 0.454
	Both	9	1.82 (1.49–2.22)	<0.001	I^2^: 46%; *p* = 0.063
Location of PNI	Extra-tumoral	2	2.28 (1.87–2.78)	<0.001	I^2^: 30%; *p* = 0.230
	Intra-tumoral	3	2.06 (1.57–2.71)	<0.001	I^2^: 0%; *p* = 0.854
	Peripheral	3	1.90 (1.10–3.25)	0.019	I^2^: 75%; *p* = 0.017
Size	>1	2	1.45 (0.81–2.60)	0.214	I^2^: 0%; *p* = 0.397
	<1	3	1.74 (1.19–2.52)	0.004	I^2^: 0%; *p* = 0.899

## Data Availability

Those data supporting the findings of this study are available within its Supplementary Materials.

## Supplementary Materials

The following supporting information can be downloaded at: https://www.mdpi.com/article/10.3390/diagnostics13213339/s1, Table S1: The characteristics of included studies and patients.
